# Modified Laparoscopic Nephron-Sparing Surgery for Large Renal Hilar Angiomyolipoma: Dual-Center Experience

**DOI:** 10.3389/fsurg.2022.901033

**Published:** 2022-06-08

**Authors:** ZeSong Yang, Fang Wang, Deng Lin, Qiuyan Li, Yun Hong, Minxiong Hu, Dahong Zhang, Liefu Ye

**Affiliations:** ^1^ Shengli Clinical Medical College of Fujian Medical University, Department of Urology, Fujian Provincial Hospital, Fuzhou, China; ^2^ Department of Clinical Medicine, Fujian Health College, Fuzhou, China; ^3^ Department of Urology, Zhejiang Provincial People's Hospital, Affiliated People's Hospital, Hangzhou Medical College, China

**Keywords:** modified, laparoscopic nephron-sparing surgery, large angiomyolipoma, renal hilum, experience

## Abstract

**Objective:**

The aim of this study is to evaluate a potential successful strategy for treating large renal hilar angiomyolipoma (RHAML) during the procedure of laparoscopic nephron-sparing surgery (NSS).

**Methods:**

The total study population includes 12 patients with large RHAMLs who underwent laparoscopic NSS in the Department of Urology of Fujian Provincial hospital and People’s Hospital of Zhejiang, ranging from January 2016 to March 2020. The perioperative variables, intraoperative procedures, and postprocedure complications were all recorded. Three months later, all patients returned to the hospital to check their postoperative recovery by reviewing the computed tomography urography (CTU) image. In the follow-up, patients were asked to have their review by CT or color doppler ultrasound every year.

**Results:**

Laparoscopic NSS was successfully performed in all patients. The average operation time was 113.33 ± 33.39 min; the intraoperative blood loss was about 137.50 ± 91.17 ml; the warm ischemia time was 25.25 ± 4.88 min; the drainage tube extubation time was 4.58 ± 2.07 days; and the hospital stay time was 6.42 ± 1.78 days. The average follow-up time was 14.58 ± 9.18 months. After 3 months, all CTU images showed an unobstructed urinary tract in the patient, and no tumor recurrence was found. In addition, no patients had renal atrophy and urine extravasation during follow-up.

**Conclusions:**

Laparoscopic NSS for RHAML is complex and technically demanding, but good surgical design and operation can achieve satisfactory surgical results. Modified laparoscopic NSS was a beneficial technique and may provide a reference for treating patients with RHAML.

## Introduction

Renal angiomyolipoma (RAML) is one of the common benign tumors of the kidney. It is usually composed of dysplastic blood vessels, smooth muscles, and mature adipose tissues of varying proportions. RAML can be part of the tuberous sclerosis complex, and approximately 80% of RAMLs occur sporadically, predominantly in women (1). Indications for RAML surgery include patients with clinical symptoms, suspicion of malignancy, large tumors (>4 cm), and women of child-bearing age (1). Since most RAMLs are benign, nephron-sparing surgery (NSS) is a preferred surgical method for it which can preserve the kidney and has apparent advantages over radical nephrectomy, especially in young patients (2, 3). Methods of NSS include laparoscopic surgery and open surgery. Laparoscopic NSS, including robotic laparoscopic NSS, has the advantages of less trauma, less bleeding, clear vision, and quick postoperative recovery, which has been widely carried out worldwide.

Renal hilar AML (RHAML) is one of the unique types of RAML. Because that RHAML has a deep anatomical location and is often surrounded by renal blood vessels and renal pelvis, NSS is difficult and risky, especially for tumors of considerable size and tumor mainly pressing the renal sinus. Although robotic laparoscopic NSS should be implemented in such cases, it is still unavailable in many departments, and the laparoscopic approach is needed. However, laparoscopic NSS of RHAML has the disadvantage of poor vision, heavy bleeding, and a high possibility of damage to the renal blood vessels and renal pelvis, which has always been a difficult point in clinical management. Most previous studies have focused on common RAMLs, with only a few analyzing laparoscopic NSS for large RHAMLs. In order to solve the problem of laparoscopic NSS for RHAML, we have summarized a set of modified laparoscopic surgical methods after numerous attempts and trial-and-error, which finally achieved favorable clinical results for us to publish.

## Materials and Equipment

### Patients

**Figure 1:**
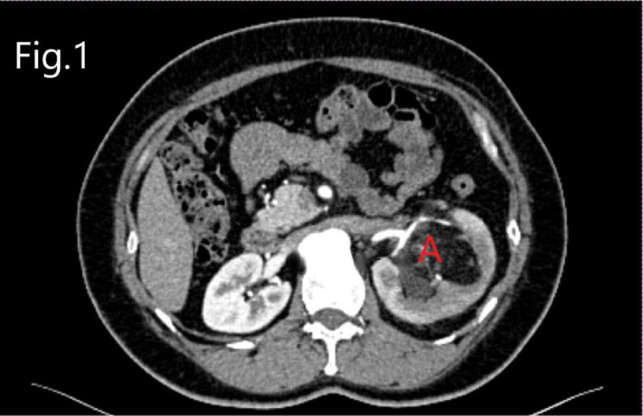
CTU image of RHAML: (**A**) tumor.

**Figure 2:**
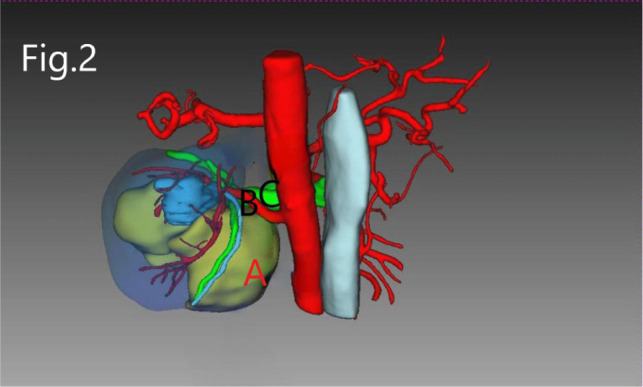
3D imaging reconstruction of RHAML: (**A**) tumor, (**B**) renal artery, and (**C**) renal vein.

From January 2016 to March 2020, 12 patients with large RHAMLs who underwent laparoscopic NSS in the Department of Urology of Fujian Provincial Hospital and People’s Hospital of Zhejiang Province were included in the study. Computed tomography urography (CTU) images were acquired for all study patients. At the same time, additional 3D imaging reconstructions were issued and conducted on seven patients (Figures 1, 2).

### Ethics Approval

The study was approved by the Ethics Committee of Fujian Provincial Hospital. Also, we certify that the study was performed in accordance with the ethical standards as laid down in the 1964 Declaration of Helsinki and its later amendments or comparable ethical standards.

### Informed Consent

Written informed consents were obtained from patients’ guardians.

## Methods

### Modified Laparoscopic Nephron-Sparing Surgery

**Figure 3:**
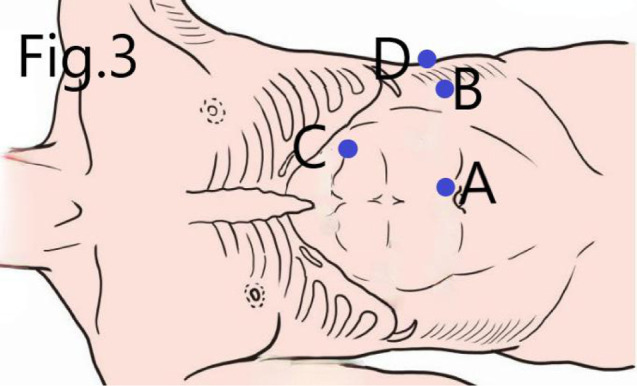
Location of trocar: (**A**) observing trocar, (**B**,**C**) operating trocar, and (**D**) auxiliary trocar.

**Figure 4:**
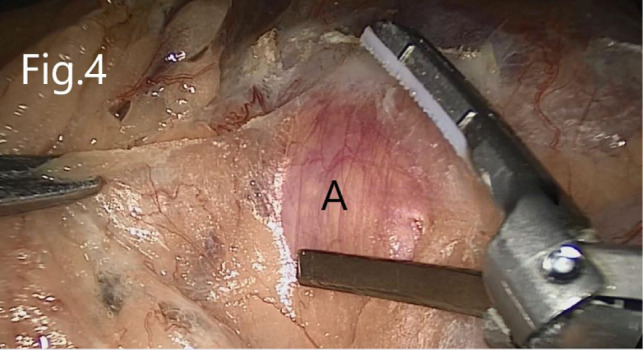
Separation of the nonvascular space between the prerenal fascia and the mesangium of the colon: (**A**) prerenal fascia.

Two experienced surgeons performed all surgical procedures using a transabdominal laparoscopic approach:

First, the patients were placed in the lithotomy position under general anesthesia. A 5F ureteral catheter was placed into the renal pelvis on the operation side over a Cook 0.38 guidewire by cystoscopy. Then, the patients were turned into a lateral position. Four trocars were placed (Figure 3), and pneumoperitoneum was established at 12–15 mmHg pressure. The peritoneum was cut along the Toldt line, and the colon and its mesangium to the inside were separated to expose Gerota’s fascia (Figure 4). If AML was on the left side, the colonic diaphragmatic ligament, splenic colonic ligament, and splenic diaphragmatic ligament were cut off. The spleen–kidney ligament and the tail of the pancreas were then separated. The spleen could naturally fall to the inside, and the upper pole of the left kidney was thus exposed.

**Figure 5:**
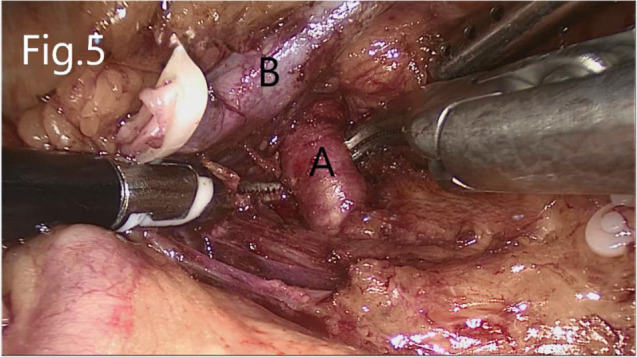
Separation of the renal artery and renal vein: (**A**) renal artery, (**B**) renal vein.

The left renal artery was found after separating upward along the abdominal aorta. If AML was on the right side, the right triangular ligament of the liver was separated, and the peritoneum was cut along the lower edge of the liver (if necessary, an additional trocar is added below the xiphoid process to push the liver upward). Next, the duodenum is separated to expose the inferior vena cava. The renal hilum on both sides was exposed after separating the kidney from the level of the lower pole of the kidney upward along the psoas muscle plane. The renal vein, renal artery, and ureter were dissected separately (Figure 5).

**Figure 6:**
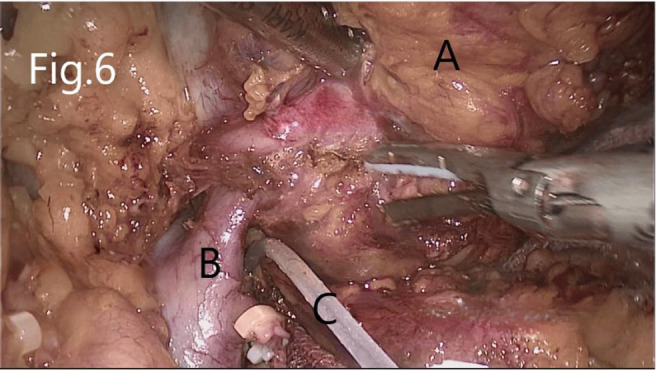
Separation of RHAML: (**A**) tumor, (**B**) renal vein, and (**C**) silicone tube that encircles the renal artery for marking.

**Figure 7:**
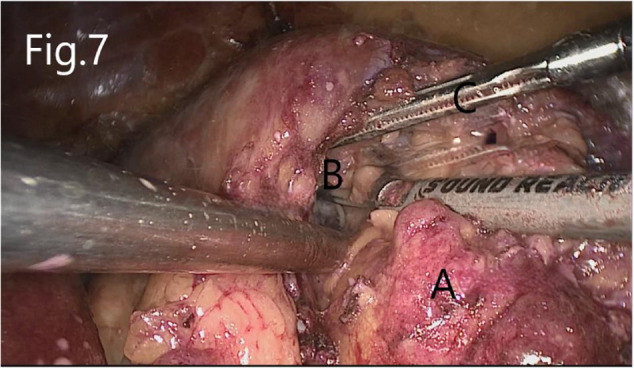
Resection of RHAML: (**A**) tumor, (**B**) renal sinus, and (**C**) surgical instruments used by the assistant to pick up the renal hilum.

**Figure 8:**
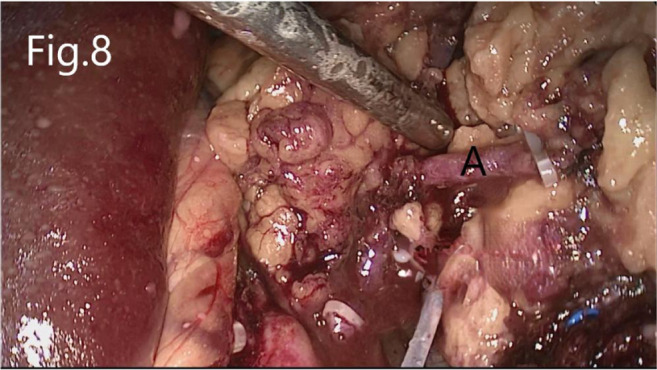
Resection of RHAML: (**A**) a small blood vessel leading to the tumor that is clamped by hem-o-lock.

**Figure 9:**
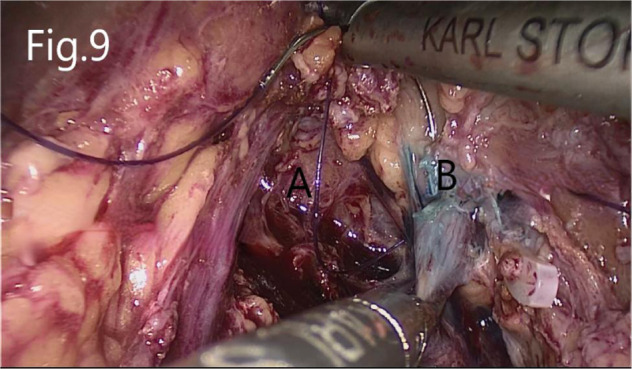
Suture of the injured renal pelvis: (**A**) renal hilum revealed after tumor removal, (**B**) injured renal pelvis.

**Figure 10:**
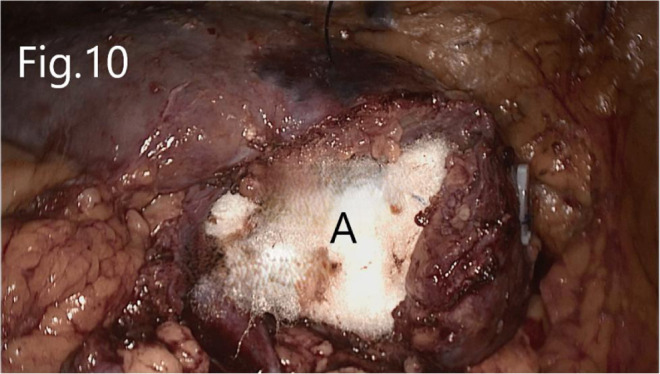
Reconstruction of renal hilum: (**A**) biological materials filled in the renal hilum.

**Figure 11:**
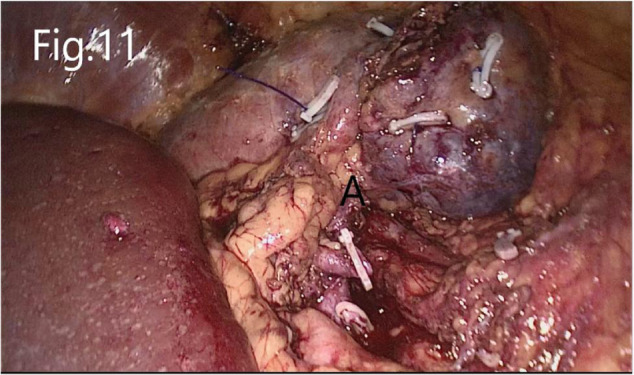
Reconstructed renal parenchyma: (**A**) there is no bleeding in the renal hilum area after the renal artery is opened.

Separate along the edge of AML to detect the boundary of the tumor (Figure 6). Then, block the renal artery. The AML was removed along with the space between the tumor and the kidney tissue (Figure 7). The small blood vessels supplying the tumor were clipped and cut off (Figure 8). If the renal pelvis ruptured, it was repaired and closed (Figure 9). After packing the bio-hemostatic material into the wound, the edge of the kidney parenchyma was sutured with barbed thread (3-0 or 2-0) (Figures 10, 11). If the renal pelvis was ruptured during tumor resection, 2-0 absorbable suture was used to suture, and a double J tube was placed.

### Follow-Up

The double J tube was removed 2 months after the operation in patients with a renal pelvic injury. All patients returned to the hospital 3 months after the operation to review the CTU and then asked to have their review by CT or color doppler ultrasound every year.

### Statistical Analysis

SPSS 20.0 software was used for statistical analysis. Measurement data were provided in a mean ± SD format.

## Results

General information about the patients included in the study is summarized in Table 1.

**Table 1:** General information about the patients.

Parameters		Value
Gender	Male, *n* (%)	3 (25%)
	Female, *n* (%)	9 (75%)
Age	<60 years, *n* (%)	11 (91.67%)
	≥60 years, *n* (%)	1 (8.33%)
	Average (years)	48.58 ± 7.82
BMI		25.79 ± 2.38
Size of AML	≥5 cm, *n* (%)	12 (100%)
	<5 cm, *n* (%)	0 (0%)
	Average (cm)	7.42 ± 1.98
History of rupture and bleeding of AML	Yes, *n* (%)	2 (16.67%)
	No, *n* (%)	10 (83.33%)
With clinical symptoms	Yes, *n* (%)	2 (16.67%)
	No, *n* (%)	10 (83.33%)
Side of AML	Right, *n* (%)	4 (33.33%)
	Left, *n* (%)	8 (66.67%)

*AML,* *angiomyolipoma; BMI, body mass index.*

Laparoscopic NSS was successfully performed in all patients. Among the study population, one patient underwent selective arterial embolization for ruptured AML 3 months prior to surgery and suffered a rupture of the renal pelvis during tumor resection due to the adhesion of the tumor to the surrounding tissues during the surgery. After proper suture, the double J tube was indwelled for 2 months. The average operation time was 113.33 ± 33.39 min, the intraoperative blood loss was about 137.50 ± 91.17 ml, the warm ischemia time was 25.25 ± 4.88 min, the drainage tube extubation time was 4.58 ± 2.07 days, and the hospital stay time was 6.42 ± 1.78 days (Table 2). Postoperative pathological results confirmed that the tumors were AML. The average post-operation follow-up time was 14.58 ± 9.18 months. After 3 months, CTU showed that all patients had unobstructed urinary tract, and no tumor recurrence was found. In addition, all patients did not have renal atrophy and urine extravasation during follow-up.

**Table 2:** Operation data.

Parameters	NSS (*n* = 12)
Operating time (min)	113.33 ± 33.39
Estimated intraoperative blood loss (ml)	137.50 ± 91.17
Drainage tube extubation time (days)	4.58 ± 2.07
Hospital stay time (days)	6.42 ± 1.78
Follow-up time (months)	14.58 ± 9.18

*NSS, nephron-sparing surgery.*

## Discussion

RHAML is one of the typical complicated renal tumors. Although, in general, very demanding in handling and expertise, the main difficulties of laparoscopic NSS are positioning, resection, and reconstruction (4, 5). Due to the RAML tumor’s fragility, it is prone to rupture and hemorrhage during the separation process and causes unclear vision, making the operation more difficult. In addition, RAML can spontaneously rupture and hemorrhage when subjected to a slight external force or even without inducement (6), and the incidence of spontaneous rupture was significantly increased in patients with a tumor whose diameter was larger than 4 cm (7, 8). Therefore, patients with a history of spontaneous rupture and bleeding could have apparent adhesion between the tumor and the surrounding tissues, making it more difficult to separate and expose the tumor tissue, thus further increasing the operation’s difficulty.

### Preoperative Preparation

#### 3D Imaging Reconstruction

RHAML is adjacent to the renal pedicle and renal pelvis. It is often difficult to clearly show the anatomical relationship between the three in conventional imaging examinations. 3D reconstruction based on CTA can accurately display the anatomical structure of the hilar, the location of the tumor, and the distance and position between the tumor and blood vessel and renal pelvis, which can significantly help the surgeon determine the surgical plan.

#### Selective Arterial Embolization

The blood vessels in renal AML are majorly constituted with immature malformed blood vessels. As a result, its blood vessels lack a complete intima, making these prone to spontaneous rupture and bleeding, which may result in hemorrhagic shock and death. Therefore, selective arterial embolization is preferable for these patients and may achieve satisfactory hemostasis. Wang et al. (9) have shown that selective arterial embolization before operation can reduce blood loss and warm ischemia time. Therefore, preoperative selective arterial embolization for larger renal hilar AML, especially those with aneurysm formation in the tumor, when performed correctly, would reduce the difficulty of the operation.

### Operation

#### Surgical Approach

In our protocol, the transperitoneal approach is a prerequisite for this surgery.

In general, laparoscopic NSS can be performed through the transperitoneal or retroperitoneal approach. The retroperitoneal approach can reduce the possibility of the abdominal organs’ injury and avoid irritation to the peritoneum. However, for RHAML, the operation space of the retroperitoneal approach is limited. Meanwhile, since the renal hilum faces the ventral and inner sides, it is hard to visually expose the renal hilum, which therefore is nearly impossible to suture and thus increases the difficulty of tumor resection and renal hilum reconstruction. In this case, for hilar tumors, a transperitoneal approach is recommended. In addition, the ureteral catheter on the surgical site must be indwelled before the operation so that the methylene blue solution can be injected during the operation to detect and repair the possible renal pelvic damage once detected.

#### Location of Trocars

An accurate trocar positioning shall help obtain a direct vision of the renal hilum and reduce the difficulty of surgery. In conventional laparoscopic NSS, observing trocar is usually positioned more laterally at the umbilicus line, facilitating the entire kidney’s visualization. Contrary to that, our location for observing the trocar is generally located near the umbilicus, which directly targets the renal hilum and facilitates the separation and resection of the tumor, and the reconstruction of the renal hilum later. An operating trocar is located at the intersection of the anterior axillary line and the horizontal line of the umbilicus. Another operating trocar is located at the lateral line of the rectus abdominis under the costal margin, in which a good suture angle can be obtained. For the auxiliary trocar, in traditional laparoscopic NSS, it is usually placed in the lateral lower abdomen for which the assistant can obtain a more comfortable position. In our approach, the auxiliary trocar is translocated at the intersection of the mid-axillary line and the horizontal line of the umbilicus, which enables the assistant to use surgical instruments to pick up the kidney and expose the renal hilum without interfering with the surgeon’s operation. Moreover, the trocar position can also be adjusted slightly intraoperative per the patient’s degree of obesity.

#### Expose of the Renal Hilum

In our protocol, ensuring that the renal hilum faces the surgeon is the basis for the operation’s success.

Separation of the kidney can increase adjustment mobility, which facilitates a better suture angle later. We recommend that the outer side and upper pole of the kidney should not be separated, which can suspend the kidney and prevent the kidney from hanging down and obstructing the view of the renal hilum. The assistant picks up the lower pole of the kidney with the surgical instrument through the auxiliary trocar, and then the renal hilum could directly face the surgeon. Since the AML outside the renal sinus often reside on the kidney in a form like a “mushroom,” it is necessary to separate the layer between the tumor and the kidney, from the head to the root, to determine the tumor boundary.

#### Tumor Resection

The renal artery is blocked after determining the tumor boundary to reduce the warm ischemic time of the kidney, which can be blocked in advance if the bleeding is severe during tumor isolation. Resection of the tumor in the renal sinus is one of the difficulties of the surgery. It is recommended to remove the tumor at the inner layer of the tumor capsule to reduce the probability of damage to the renal arteries, veins, and renal pelvis. The incision is made along the upper pole of the tumor, and then the assistant uses the surgical instrument to pick up the upper edge of the surgical section to get a clear exposure. During the resection, an ultrasonic knife was used to cut the cord-like tissue. The blood and the tumor tissue in the capsule are sucked away with an aspirator to keep a clear view. Do not separate blindly when the visual field is unclear, and the ultrasound knife should not clamp the tissue too deeply to avoid damage to vital structures. The larger blood vessels in the tumor are cut off after being clipped by hem-o-lock. Titanium clips are used to temporarily clamp the vessels of the kidney if it is injured, which is sutured and reconstructed after the tumor is removed. A huge rupture of the renal pelvis is closed after inserting a double J tube. The capsule of the tumor is treated by electrocautery to avoid tumor recurrence.

#### Suture and Rreconstruction of the Renal Hilum

In our protocol, “Unobstructed suture parallel to the blood vessel” is the key to success.

After removing the tumor, methylene blue can be injected through the pre-indwelling ureteral catheter to determine whether there is a renal pelvic injury and whether a proper suture is done. Minor bleeding in the renal sinus does not need treatment, while severe bleeding could be stopped by sutures. Since the renal hilum contains renal arteries, veins, and renal pelvis, the sutures in the renal sinus should be cautious to avoid injury to the important structure’s upside. Therefore, we take an “Unobstructed suture parallel to the blood vessel” approach to deal with this issue: (1) The tract of the access needle must be parallel and far away from the vascular structure in the renal hilum, like a sector interval. In this case, when the thread is closed, the blood vessel will not be compressed. (2) The suture depth should be limited to avoid damage to renal arteries, veins, and renal pelvis. Once the wound is filled with suitable materials (such as absorbable hemostatic gauze), 2-0 barbed sutures can be used to close the outer peripheral renal parenchyma or the lips on both sides of the kidney hilum to compress and stop the bleeding. Our experience is that applying a hem-o-lock clip to the source of the thread on the surface of the kidney with each stitch could increase support and prevent the suture from cutting the kidney.

### Postoperative Treatment

During the recovery period, the caretaker must pay close attention to the amount and color of the drainage fluid. Patients with extensive drainage and suspected urine leakage should be checked with the drainage fluid for creatinine. If the renal pelvis is sutured and the double J tube is indwelled during the operation, the indwelling time of the drainage tube and urinary tube can be appropriately extended. If a renal pelvic injury is not found during the operation, the double J tube on the affected side should be indwelled in time after the operation. In addition, if the drainage volume suddenly increases and the fluid appears bright red in correspondence with simultaneous hemoglobin decreases, renal bleeding should be considered and thus requires timely intervention, such as selective arterial embolization or emergency surgical exploration.

### Treatment of Special RAML Patients

#### Obese Patients

Patients with obesity usually have more fat around the kidneys, in which the intraoperative exposure is often not satisfactory. For these personals, the operation holes can be appropriately biased to the outside, and the fat around the kidney during the operation should be removed. A satisfactory visual field must be obtained before the tumor resection and the reconstruction of the kidney hilum to prevent any sequelae.

#### Patients With Previous Renal AML Rupture and Bleeding

AML tumors with rupture and experienced hemorrhage in the past are often attached to the surrounding tissues, making it difficult to dissociate. For these personals, the surgeon’s patience would be vital while cutting sharply to separate the kidney and the tumor’s surface while preserving essential structures of the renal hilum. The operating surgeon should not hesitate to change to open surgery once the enormous obstruction is to be found following the protocol of conventional laparoscopy NSS.

## Conclusion

Laparoscopic NSS for RHAML is complex and technically demanding, but good surgical design and operation can achieve satisfactory surgical results. Modified laparoscopic NSS was a beneficial technique and may provide a reference for treating patients with RHAML.

## Data Availability

The original contributions presented in the study are included in the article/supplementary material, further inquiries can be directed to the corresponding author/s.

## References

[1] Liu X Ma X Liu Q Huang Q Li X Wang B. Retroperitoneal laparoscopic nephron sparing surgery for large renal angiomyolipoma: our technique and experience. A case series of 41 patients. Int J Surg. (2018) 54:216–21. 10.1016/j.ijsu.2018.04.043 29723675

[2] Wein AJ Kavoussi LR Partin AW Peters CA. Angiomyolipoma. In: Kavoussi LR, Partin AW, Peters CA, editors. 11 ed. *Campbell-Walsh urology*. Elsevier (2016). p. 1306–9.

[3] Ljungberg B Hedin O Lundstam S Warnolf A Forsberg AM Hjelle KM. Nephron sparing surgery associated with better survival than radical nephrectomy in patients treated for unforeseen benign renal tumors. Urology. (2016) 93:117–23. 10.1016/j.urology.2016.01.037 27017902

[4] Dulabon LM Kaouk JH Haber GP Berkman DS Rogers CG Petros F. Multi-institutional analysis of robotic partial nephrectomy for hilar versus nonhilar lesions in 446 consecutive cases. Eur Urol. (2011) 59:325–30. 10.1016/j.eururo.2010.11.017 21144643

[5] Di pierro GM Tartaglia N Aresu L Polara A Cielo A Cristini C. Laparoscopic partial nephrectomy for endophytic hilar tumors: feasibility and outcomes. Eur J Surg Oncol. (2014) 40:769–74. 10.1016/j.ejso.2013.11.023 24370283

[6] Jinzaki M Silverman SG Akita H Nagashima Y Mikami S Oya M. Renal angiomyolipoma: a radiological classification and update on recent developments in diagnosis and management. Abdom Imaging. (2014) 39:588–604. 10.1007/s00261-014-0083-3 24504542 PMC4040184

[7] Vallejo BJ Herrera TE Domenech CA Lafuente PM de Ramírez TI Robles MJ. Renal angiomyolipoma: presentation, treatment and results of 20 cases. Actas Urol Esp. (2008) 32:307–15. 10.1016/S0210-4806(08)73835-X 18512387

[8] Takebayashi S Horikawa A Arai M Iso S Noguchi K. Transarterial ethanol ablation for sporadic and non-hemorrhaging angiomyolipoma in the kidney. Eur J Radiol. (2009) 72:139–45. 10.1016/j.ejrad.2008.06.017 18632236

[9] Wang D Li HZ Ji ZG Effectiveness and safety of laparoscopic enucleation combined with selective arterial embolization for renal angiomyolipoma. Cancer Biomark. (2017) 19:177–83. 10.3233/CBM-160501 28387661 PMC13020718

